# Evaluating the Influence of Row Orientation and Crown Morphology on Growth of *Pinus taeda L*. with Drone-Based Airborne Laser Scanning

**DOI:** 10.34133/plantphenomics.0264

**Published:** 2024-10-23

**Authors:** Matthew J. Sumnall, David R. Carter, Timothy J. Albaugh, Rachel L. Cook, Otávio C. Campoe, Rafael A. Rubilar

**Affiliations:** ^1^ Department of Forest Resources and Environmental Conservation, Virginia Polytechnic Institute and State University, Blacksburg, VA 24061, USA.; ^2^ Department of Forestry and Environmental Resources, North Carolina State University, Raleigh, NC 27695, USA.; ^3^ Universidade Federal de Lavras, Lavras, MG, Brazil.; ^4^ Cooperativa de Productividad Forestal, Departamento de Silvicultura, Facultad de Ciencias Forestales, Universidad de Concepción, Concepción, Chile.; ^5^ Centro Nacional de Excelencia para la Industria de la Madera (CENAMAD) - ANID BASAL FB210015, Pontificia Universidad Católica de Chile, Santiago, Chile.

## Abstract

The tree crown’s directionality of growth may be an indicator of how aggressive the tree is in terms of foraging for light. Airborne drone laser scanning (DLS) has been used to accurately classify individual tree crowns (ITCs) and derive size metrics related to the crown. We compare ITCs among 6 genotypes exhibiting different crown architectures in managed loblolly pine (*Pinus taeda L.*) in the United States. DLS data are classified into ITC objects, and we present novel methods to calculate ITC shape metrics. Tree stems are located using (a) model-based clustering and (b) weighting cluster-based size. We generated ITC shape metrics using 3-dimensional (3D) alphashapes in 2 DLS acquisitions of the same location, 4 years apart. Crown horizontal distance from the stem was estimated at multiple heights, in addition to calculating 3D volume in specific azimuths. Crown morphologies varied significantly (*P* < 0.05) spatially, temporally, and among the 6 genotypes. Most genotypes exhibited larger crown volumes facing south (150° to 173°). We found that crown asymmetries were consistent with (a) the direction of solar radiation, (b) the spatial arrangement and proximity of the neighboring crowns, and (c) genotype. Larger crowns were consistent with larger increases in stem volume, but that increases in the southern portions of crown volume were consistent with larger stem volume increases, than in the north. This finding suggests that row orientation could influence stem growth rates in plantations, particularly impacting earlier development. These differences can potentially reduce over time, especially if stands are not thinned in a timely manner once canopy growing space has diminished.

## Introduction

The spatial arrangement of the forest canopy and architecture of individual trees is determined by a number of factors, such as the availability of resources, the competition of neighboring individual tree crowns (ITCs), and the presence and size of canopy gaps (e.g., [1–5]). Ford [6] concluded that the growth of these individuals would be determined by the size, shape, and efficiency of its crown, and represent an expression of resource acquisition [7] (e.g., photosynthetic potential, evapotranspiration, and respiration). Dutilleul et al*.* [8] stated that tree crown characteristics important for light interception efficiency are its geometric structure, branching pattern complexity, and the amount of leaves. Tree crown structure has been characterized by using morphological measures of crown dimensions (e.g., volume, surface area, height, and height of crown base; [9,10]). Geometric models for specific species have been developed using these crown inputs to simulate future growth, including stem volume (e.g., [11]). Many forest modeling and yield forecasting methods (e.g., [12,13]) incorporate measurements of ITC size [10,11] and neighborhood competition metrics [14,15]. These methods assume a simplified crown shape, such as a symmetrical ellipsoid, and do not consider the presence of canopy gaps.

The conceptualization of symmetrical tree crowns potentially underestimates canopy light availability, as trees can often exhibit plasticity in terms of asymmetric growth, and complex branching structures, in order to occupy space that results in effective light interception [16]. Existing crown geometric models simplify crown geometric shapes to ellipsoid, paraboloid, cone, partial spheroid, and their combinations [11,17]. These models do not represent the spatial heterogeneity of trees with regard to the actual space occupied by tree crowns [18]. Pretzsch [9] highlights that mixed species canopies, when their architectures are complementary, enable a denser canopy, where some species develop more complex asymmetrical and overlapping crowns [16]. Thus, crown complexity may be considered as a measure of plasticity and adaptability to the environment. In addition to stem density and species effects, factors such as terrain, slope, and directionality of solar radiation can influence crown asymmetry [19,20]. This plasticity in crowns has been hypothesized as a response to the local environment and foraging for access to light [21,22]. Thus, the priority of the crown’s directionality of growth may be an indicator of how aggressive the tree is in terms of foraging for light. The proximity and size of competitors will influence individual tree stem growth and form in addition to crown shape [23].

There are few studies investigating the effects of the spatial arrangement of tree stems with regard to productivity; however, Fraver et al. [24] noted that their influence was significant. York et al. [25] noted differences in tree growth relative to the individual’s proximity and direction to gaps or, more specifically, access to light for a number of coniferous species. The effects of row orientation have been explored with orchard or row crops (e.g., [26,27]). A large amount of sunlight will shine directly on the gap between rows and not be intercepted by the plants. For some crops, a north-to-south row orientation has been found to be advantageous (e.g., [28]). From a forest production point of view, the priority is the productivity of the stem. The influence of planting row orientation on pine plantation productivity has seen limited exploration in the research literature, particularly concerning loblolly pine (*Pinus taeda*) (e.g., Amateis et al. [29]). Assessing these individual tree-level metrics over large areas represents a substantial investment in terms of time and effort.

Accurately measuring ITCs with potentially asymmetric 3-dimensional (3D) shapes presents a number of logistical challenges. Crown horizontal projection is typically measured from the ground in 8 directions from the stem to the canopy drip line [30–33]. This can only represent a tree crown as a simplified shape. More detailed measurements of crown exterior shape can be acquired in 3D by using a tachymeter or total station [34,35]. Recently, terrestrial laser scanning (TLS) has been used, where the tree crown is scanned from multiple directions. Tree crown geometry metrics are often derived from fitting simple geometric shapes encompassing the tree classified returns [16] or via the manual determination of the horizontal footprint [32]. Such approaches are time consuming and spatially limited and may not fully represent the complex shape of the individual crown.

Remote sensing technologies, specifically airborne laser scanning (ALS), has been used to study the changes of vegetation through time, with many uses in agriculture (e.g., [36]). ALS has been used to accurately classify ITCs and derive size metrics such as crown horizontal projection area, crown length, and geometric volume (e.g., [37,38]) over large spatial extents. These crown metrics were significantly related to estimates of stem diameter and volume. Kato et al. [35] demonstrated an approach to create a 3D surface wrapped around the exterior of classified returns for complex and irregular tree crowns of multiple tree species.

Unmanned aerial vehicle or drone laser scanning (DLS) data can generally provide a greater pulse density in comparison to ALS from piloted aircraft due to operating altitude (e.g., [39]). A high pulse density and high, off-nadir, scan angles have allowed for the development of methods for the automatic classification of tree stems [40]; however, point density is often too low to estimate stem size directly. This capability potentially overcomes issues with ALS in sufficiently penetrating the tree crown and providing data from the lowest portions of the tree crown (e.g., [40–44]). Monitoring vegetation change over time via remote sensing technology has seen wide use from satellite multispectral imagery (e.g., [45,46]). Song et al. [47] demonstrated that accurate 3D crown shape estimates could be accomplished through airborne drone-acquired imagery, specifically derived from structure from motion, which were sensitive to genetic variation in *Pinus elliottii* forest. The use of time-series ALS data, in many situations, can provide improved change detection results compared to passive imaging approaches [48]. The ability of ALS and DLS to provide returns from within and below the canopy surface potentially allows us to quantify 3D morphological shape and therefore change, provided any differences in acquisition are accounted for [49]. The detection of ITCs and their comparison through time (e.g., [50,51]) allows the comparison of 3D crown geometry over time over larger spatial extents and populations than previously implemented.

Given the importance of tree crowns to tree and system-wide productivity [38] and DLS scanning’s relatively underutilized capabilities in characterizing crown shape, we explored the ability of DLS to track ITC morphology over time. The evaluation of ITC shape and complexity has been evaluated previously using TLS scans (e.g., [8,52]). ITC growth direction is typically evaluated relative to the stem location (e.g., [53]), given that the expected lower pulse density of DLS acquisitions in comparison to TLS and the difficulty in classifying features, such as the stem or branches [40], present a problem. Research has been used to classify portions of the tree stem in previous research, with sufficient point density ([40,42,44]); thus, a reference of crown shape can be made to the stem’s horizontal location. As noted above, ALS can be used to characterize complex exterior shapes of tree crowns (e.g., [35]). Zhou et al. [37] utilized 3D alphashapes to characterize tree crowns with convex features, and thus, direction and distance from the stem could be calculated to the exterior of this shape.

While ITC volume is important for biomass production (e.g., [54]), the architecture of the crown represents a number of environmental factors, such as competition, wind, and water availability [55], which is reflected in branching pattern and exterior shape. Thus, evaluating the complexity of this shape may help us more understand tree function and form. Methods exist for the evaluation of 3D shape surface morphological complexity (e.g., [56]), often developed for non-forestry-related applications. The 3D surface of the object is expressed as a 3D mesh consisting of interconnected triangular polygons fit to the exterior of the object, for example, a 3D scanned tooth. The orientation patch count rotated (OPCR) [56–58] is one such example. The OPCR is defined as the number of regions on a surface (“patches”), where adjacent polygons within a patch all face the same direction (i.e., similarly angled normal vectors when projected on the horizontally orientated plane).

High pulse-density DLS data are useful in providing spatially explicit metrics related to ITC size. A means of evaluating the characteristics of the 3D crown shape through time may improve our understanding of crown development and how, in turn, stem size is affected. Therefore, in this analysis, we propose a novel approach to evaluate ITC morphology over time for the assessment of asymmetric crown development and crown shape complexity within an experimental loblolly pine (*P. taeda* L.) forest, which included 6 different genotypes exhibiting differences in crown architectural traits [59], where each tree has been mapped using Global Positioning System (GPS) and monitored since planting. We would use the increased point density of the DLS to identify individual stem location and the generated metrics of the 3D crown shape relative to this. Specifically, we developed an approach to estimate the horizontal distance of stem center to the crown exterior for multiple heights and azimuths to quantify growth direction and crown presence. In addition, we would further segment 3D shapes using planes to quantify crown volume in specific azimuth ranges. We would also further evaluate the differences in the 2 row orientations that existed in the trial location. Our specific objectives were as follows:

1. Implement a method to classify ITCs from 2 temporally distinct DLS acquisitions and validate it against field stem mapped data in stands with varied crown architectures;
2. Develop an approach to calculate 3D shapes encompassing each of the classified ITCs from the DLS point cloud;
3. Derive metrics from each of the ITC 3D shapes related to directionality of growth relative to the tree stem location (asymmetry) and crown shape complexity, and then use these as statistical model inputs to evaluate their influence on growth changes over time;
4. Statistically analyze any differences in crown shape related to prevalent solar direction and row orientation.

## Materials and Methods

### Study sites and field sampling

**Fig. 1.**
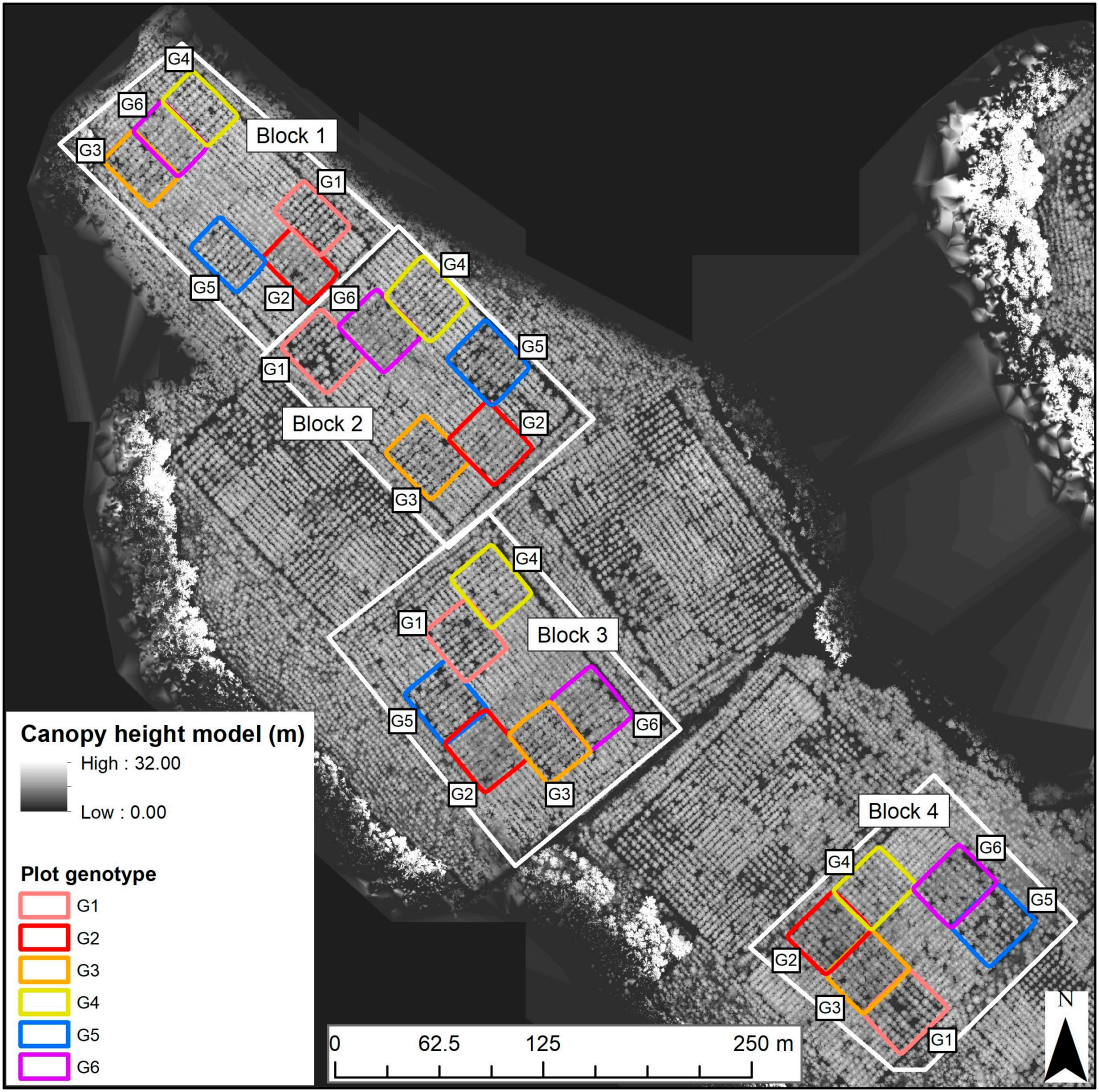
The 24 field plots installed within 4 block (replication) layouts at the Reynolds Homestead study site. The locations of each of the 6 genotypes (G1 to G6) are annotated. The canopy height model was derived from drone laser scanning (DLS) data acquired in 2017.

Our study site was located within Reynolds Homestead (RH), in the Virginia Piedmont, United States (36°38′33.04″N, 80°9′16.77″W). Local terrain slope within the site varies from 2° to 15°. The purpose of the research installation was to assess the interactions between genotypes and silvicultural intensity [59,60]. The study site is illustrated in Fig. 1.

The RH site was established in 2009 with 4 replications (blocks) of 6 loblolly pine genotypes. Complete understory weed control and fertilization as needed were applied for the first 3 growing seasons. Overall understory presence was minimal in both 2017 and 2021. There were 24 field plots, with 6 plots per block (one of each genotype). Block 1 had 63 trees per plot (7 × 9, for 6 plots), and the remaining blocks 2, 3, and 4 had 81 trees per plot (9 × 9, for 18 plots). Each of the plots had a planting density of 618 trees per hectare (4.42 × 3.66 m). Thus, for each genotype, there were 4 plots and a total of 306 individual trees each. A total of approximately 1,836 individual trees were installed. The genotypes maximized within group homogeneity and ensured different tree crown structures, among the 6 genotypes tested. These genotypes included 4 clones: 2 with narrow crowns (G1 and G3) and 2 with wide crowns (G2 and G4). The remaining 2 genotypes consisted of one control-pollinated family (G5) and one open-pollinated (G6) family [59]. The research presented by Yáñez et al. [59] found that the crown genotypes exhibited different attributes (crown diameter and height to the live crown). Differences were found in lower crown branch mortality between broad-crown clones, thus suggesting crown physiology differences between clones.

Blocks 1, 2, and 3 had the same row directions, where each row (4.42-m spacing) followed an azimuth of ~135°. Block 4 followed an azimuth of ~45°. We therefore recoded blocks 1, 2, and 3 as class 1 and block 4 as class 2.

Each of the tree’s GPS locations was recorded at the time of planting in 2009. All trees (in both sites) were measured in January of 2017 and 2022. For all trees, we measured diameter at breast height (DBH; at a height of 1.37 m). For the central 25 (5 × 5) trees in each of the 24 plots, we measured tree top height. A Vertex hypsometer was used to measure top height with an expected measurement error of 0.2 to 0.3 m [61]. Again, for the central 25 tree stems, 2 crown horizontal diameter measurements were acquired: (a) within and (b) across planting rows. These measurements were made at ground level with a tape held from crown edge to edge. The crown edge was considered the perimeter of the visible crown from the ground. Merchantable stem volume was calculated using the unthinnned, outside-the-bark model from Tasissa et al. [62]. This model, modified for metric units, is expressed as:

$$\begin{array}{c} \begin{array}{c} {\mathrm{SV}}_{ob}=\left(0.21949+0.00238\times {\left(D\times \beta_{1}\right)}^{2}\times \left(H\times \beta_{2}\right)\right)\times \beta_{3}\\ \;\beta_{1}=0.393701\\ \;\beta_{2}=3.28084\\ \;\beta_{3}=0.0283168 \end{array} \end{array}$$ (1)

where *SV_ob_* is the total outside bark volume (in cubic meters), *D* is DBH (units in centimeters), and *H* is total tree height (units in meters). The coefficients *β_1_* and *β_2_* convert metric units to imperial, and vice versa for *β_3_*. The difference between DBH and *SV_ob_* for the years 2017 and 2021 was then calculated.

### Drone laser scanning data acquisition and preprocessing

In 2017, DLS data were acquired in April for the RH site. A Vapor-35 platform was used (AeroVironment, Simi Valley, CA, USA) carrying a YellowScan Surveyor Core LiDAR unit (Monfeerier-sur-Lez, France). This unit consisted of a Velodyne VLP-16 laser scanner (Velodyne, San Jose, CA, USA) and a GNSS-inertial Trimble APPLANIX APX-15 (Trimble, Richmond Hill, ON, Canada). The specifications are summarized in Table 1. Preprocessing and strip adjustment were conducted before DLS data processing in the YellowScan CloudStation software. Ground control used a real-time kinematic geographic positioning system (RTKGPS; Topcon GR-3) with precision ≤10 cm and positional accuracy ≤5 cm.

**Table 1.** Drone laser scanning acquisition characteristic summary

Specification	Description	
Year	2017	2021
Laser wavelength	905 nm	905 nm
No. returns recorded per pulse	≤2	≤6
Point density	312–498 pulses m^−2^	700–1,011 pulses m^−2^
Scan angle	≤60°	≤85°
Flight altitude	50 m^a^	60 m^a^
Flight line overlap	50%	80%
Pulse repetition rate	300 kHz	100 kHz

^a^ Relative to take-off location.

In 2021, the RH site was reflown with a different DLS system, during July. The DJI M600 hexacopter platform (DJI Technology Co. Ltd., Shenzhen, China) provided the platform. The sensor was a Riegl MiniVux1 LiDAR scanner (RIEGL Laser Measurement Systems GmbH). This also incorporated an APPLANIX APX20 GNSS-inertial measurement unit (Trimble, Richmond Hill, ON, Canada). The specifications are summarized in Table 1. Preprocessing and strip adjustment were conducted before DLS data processing in the RiPROCESS software (RIEGL Laser Measurement System GmbH, Horn, Austria). Ground control was provided by a RTKGPS (Spectra SP80) with a post-processed positional accuracy ≤0.3 cm.

All preprocessing and analyses were conducted in the R software (version 4.3.1.) ([63]). Table 2 summarizes all packages used in the analysis. We preprocessed the DLS data by initially classifying and removing potential noise points, using the isolated voxel filter ([64,65]). We used the following settings, which were determined visually after several permutations. This method functions by creating a 3D matrix encompassing the extent of the point cloud, where voxels were cubic with a side length of 1 m. Returns were classified as noise if only one return was detected within a 3 × 3 × 3 m search kernel. The cloth simulation filter [66] method was used to classify ground returns. Aboveground return heights were calculated by first creating a surface from ground-classified returns, using a triangular irregular network; this height was then subtracted from all non-ground classified returns.

**Table 2.** Summary of R packages used in this analysis

Package name	Version	Reference
dplyr	1.1.0	[93]
lidR	4.0.2	[64,65]
lidRplugins	0.4.0	[69]
data.table	1.14.8	[94]
sf	1.0.9	[95]
terra	1.7.3	[89]
dbscan	1.1.11	[96]
MASS	7.3.58.1	[97]
soilphysics	5.0	[98]
deldir	1.0.6	[99]
ggplot2	3.4.1	[100]
stringr	1.5.0	[101]
mclust	6.0.0	[102]
alphashape	2.5	[103]
alphashape3d	1.3.2	[72]
rgl	1.0.1	[104]
doolkit	1.42.2	[74]
circular	0.4.95	[75]
lme4	1.1.31	[77]
lmerTest	3.1.3	[79]
emmeans	1.8.4.1	[78]
MuMIn	1.47.5	[80]
car	3.1.2	[105]

### Individual tree crown processing

We implemented 2 ITC delineation approaches, summarized below. The results of each will be compared to field data with regard to commission/omission error and crown horizontal diameter estimate accuracy. Only the approach that provided the most accurate results, in terms of crown horizontal size, would be used for further analysis.

We implemented the ITC delineation method as described by Sumnall et al. [38]. The ITC delineation process was conducted for each of the DLS acquisitions (in 2017 and 2021). No changes to the method were made with regard to the datasets from either acquisition. In summary, initial ITC center coordinate markers were located using a local maximum search of a raster layer representing a 2D vertical occupancy index from vertically aggregated 0.2 × 0.2 × 0.2 m voxels. ITC horizontal extents were established using the marker-based approach defined by Silva et al. [67] using a 0.1 × 0.1 m canopy height model (CHM). The non-ground classified returns were reclassified into ITC objects using this layer. All returns, below 1-m height aboveground, were considered as understory vegetation and removed.

A second ITC delineation method was applied to both datasets using the PTrees method [68] to classify the point cloud directly, as implemented in lidRplugins [69]. This approach was implemented to ensure that segmentation boundaries would not potentially be influenced by the CHM pixel size. The method uses multi-scale and dynamic segmentation approaches based upon *k*-nearest neighbors and convex hulls. We initially tested a number of *k*-nearest neighbors to use for a 10% subset of the dataset (both years). Given the high-point density for our DLS datasets, *k*-nearest neighbors to use were set to 1,000; all points below 2 m were excluded and could not form apices or increase convex-hull sizes.

The DLS point cloud was subset by the ITC classification, and metrics related to the ITCs location size were calculated. We assessed each ITC separately. Total or top height was defined as the tallest return height associated with the ITC classification. The mean average of ITC horizontal (*X* and *Y*) coordinates of returns in the top 50% of height was considered as the center position of the crown.

**Fig. 2.**
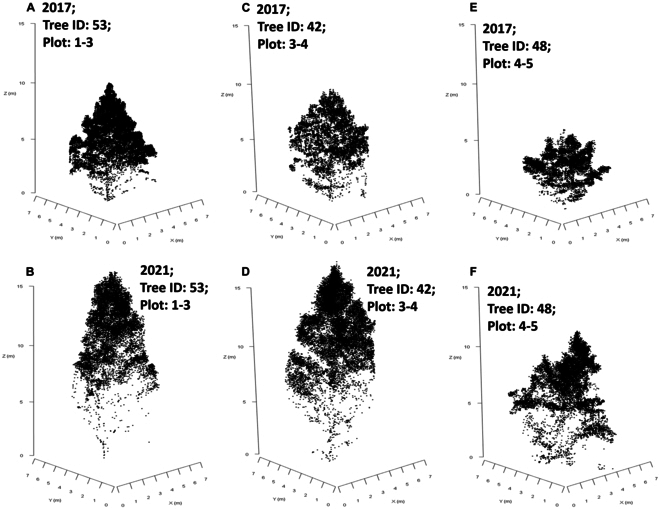
Individual tree crown point clouds classified from the DLS data and spatially matched for 2017 and 2021. (A and B) Tree number 53 from plots 1 to 3 (block 1; genotype 3—narrow crown) for data acquired in 2017 and 2021, respectively. (C and D) Tree number 42 from plots 3 and 4 (block 3; genotype 4 —wide crown) for data acquired in 2017 and 2021, respectively. (E and F) Tree number 48 from plots 4 and 5 (block 4; genotype 5—control pollinated) for data acquired in 2017 and 2021, respectively.

We paired ITCs using these central coordinates, from the 2 acquisition dates, with their closest horizontal neighbor. Duplications of ITC unique identifiers were not permitted. If the distances between pairs of center coordinates were more than 2 m, ITCs could not be paired. This process was repeated to link paired ITC geometric centers to planting GPS locations. Examples of the differences in individual tree point clouds (for classified and paired ITCs) for 2017 and 2021 are illustrated in Fig. 2, in addition to visible variation in laser penetration to lower portions of the crown.

**Fig. 3.**
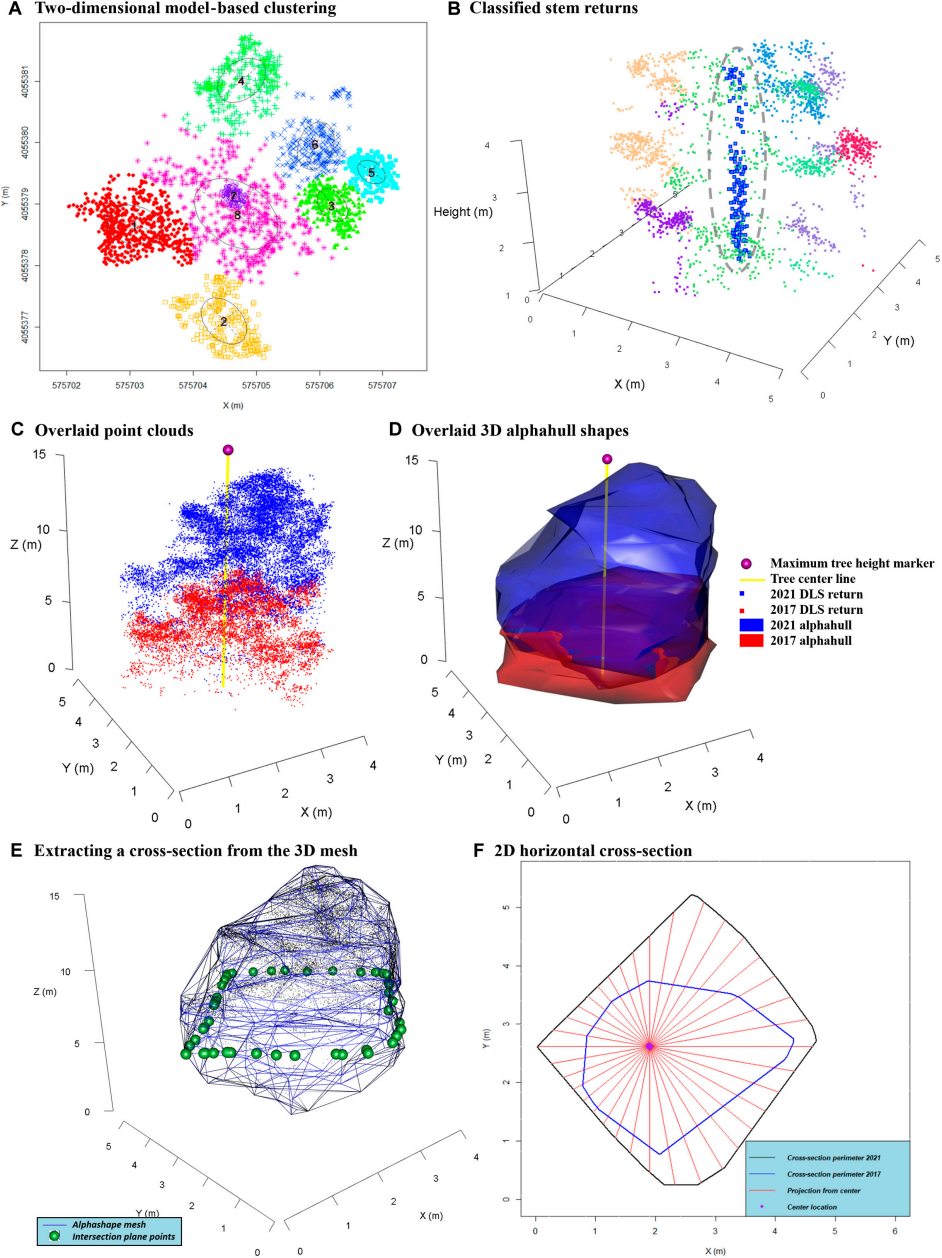
All plots represent a point cloud for a portion of a single tree (lowest 50% of total height with heights below 1 m removed). Model-based clustering (A) is applied to the data, where clusters are colored randomly. Weights are applied, and a likely stem cluster is classified in 3D space (here, cluster 7). Returns likely belonging to the stem are illustrated in (B) (highlighted in blue or dashed gray line). Two overlaid point clouds (C) and overlaid 3D alphashape shapes (D) for a single tree in 2017 and 2021. The red 3D point cloud (C) and shape (D) represent the tree crown scanned in 2017 and, likewise, in blue for 2021. The yellow lines represent the estimated stem location, and the ball represents tree top height. An example of estimating crown projection directions and distance from the stem center. A cross section is identified on the ITC alphashapes 3D mesh around the point cloud (E) via a plane (F), in this example at a height of 6 m. The green spheres in (E) represent the outline of the plane shown in (B). The resulting cross-section is converted into a standard 2D polygon (F), and from the estimated stem location, the projected distance to the polygon edge is calculated every 10°.

For the purposes of defining a reference location for crown horizontal growth, it was necessary to estimate the stem location. We expected that the stem would be most identifiable in the bottom half of total tree height. We assumed the stem to be straight and vertical. In order to best estimate crown horizontal projection, it was necessary to classify those ITC returns most likely to be from the tree stem; thus, returns below 50% of the total height of the ITC returns were subset. A Gaussian mixture model-based clustering approach was applied to this 3D point-cloud subset (X, Y, and Z coordinates) where the optimum number of clusters was determined automatically using the entropy method documented by Baudry et al. [70], as implemented in the mclust package (an example is provided in Fig. 3A). Any cluster containing less than 5 returns was ignored. If all of the clusters were too small, the mean average of horizontal (*X* and *Y*) coordinates of canopy points (all returns above 50% of top height) was assumed to be tree center horizontal coordinates.

Four metrics were computed to characterize the size of each potential stem cluster identified in the previous step: (a) the cluster center (mean *X* and *Y* coordinates) horizontal distance to crown geometric center, (b) the difference in height value range (maximum-minimum), and (c) horizontal convex hull area. The fourth variable was calculated as a sequence of 1-m tall height bins from 1 m to 50% of the total tree height, where, if one or more returns were incident within each height bin, a value of one was recorded, and zero for no returns. A sum of these values divided by the total number of bins comprised the fourth variable: (d) the proportion of vertical occupancy. Each of these metrics were rescaled, with respect to all of the clusters, to be between a value of 0 and 1 using the following:

$$p_{\mathrm{var}}=\frac{\left(x-x_{\min}\right)\times \left(1-0\right)}{\left(x_{\max}-x_{\min}\right)}$$ (2)

where *p_var_* is the rescaled cluster metric and *x* is the individual cluster metric value. The notation *x_max_* and *x_min_* represent the maximum and minimum value of all clusters for that metric, respectively. The aforementioned metrics 1 and 3 were inverted (i.e., *p* = 1 − *p*) to prioritize clusters closest to the center and smaller horizontal areas, respectively.

We expected branches and foliage beneath the height to the live crown to be projected horizontally (e.g., [71]) and to have small vertical extents. Therefore, we assumed a stem cluster to occupy a larger height range than that of other clusters and to return consistently across this vertical range. We then developed the following weighting equation based on a 10% sample of classified clusters across our 2017 and 2021 DLS datasets. Each of the clusters was then assigned a weight value (*W*) using the following equation:

$$W=p_{1}+\left(p_{2}\times 2\right)+p_{3}+\left(p_{4}\times 2\right)$$ (3)

where *p_1_* … *p_4_* represent the 4 cluster metrics. Those clusters with the largest weight value represented the cluster containing returns from the stems (illustrated in Fig. 3B). A mean average of the stem cluster return *X* and *Y* coordinates were then used to represent the stem location, rather than the ITC return geometric center. For the paired ITCs, the coordinates of the stem cluster with the largest number of returns for either year were given priority; otherwise, the coordinates were averaged (mean). The accuracy of stem estimated locations was assessed against field GPS coordinates.

In order to approximate the visual outline of the tree crowns, an alphashape 3D mesh was calculated for each complete ITC point cloud, separately for both dates, using the ashape3d function within the alphashape3d package [72,73]. The same α value, which was set as 1 m (or *α* = 1), was used for all ITCs. The *α* value can be defined such that an edge of a sphere, with a radius of 1/*α*, can be drawn between any 2 diametrically opposed edge members of a set of points and still contain all the points within the sphere. An example of alphashapes created for both 2017 and 2021 acquisitions for a single tree is provided in Fig. 3C and D. Alphashape volume and surface area were calculated.

### Alphashape crown metric calculation

#### 2D crown growth directionality

We developed the following approach in order to determine if there is a prevalent direction of crown growth. For all ITCs (*n* = 1,836) across both 2017 and 2021, we calculated a horizontal cross-sectional polygon, vertically every 0.5 m, from 1 m aboveground to the total height of the tree (e.g., 1.0, 1.5, 2.0, 2.5 …*n*) from the alphashape 3D mesh (Fig. 3E). If the crown cross-section polygon intersected with the tree center location coordinates, then directional distance from the center was calculated. If there was no intersection of the 3D objects at that height (e.g., below the live crown), no directional distance was recorded for that cross-section. Distance from stem center was calculated for every 10° of azimuth, beginning with an azimuth of 0°, illustrated in Fig. 3F. For each height, there were 36 azimuth classes and distances from the stem center to the crown horizontal exterior.

For each ITC, we summed all cross-section crown horizontal distances for each of the 36 azimuths (i.e., every 10°) for all height classes (Fig. 3E and F). In order to measure the 2D crown temporal change, with regard to directionality, we calculated 5 metrics per ITC crown temporal pair. The first 2 were as follows:

1. The largest cumulative crown 2D projection azimuth for 2017: The azimuth with the largest crown distance from the stem summed across all height classes represented the crown’s most prevalent growth direction.
2. The largest cumulative crown 2D projection azimuth for 2021: The azimuth with the largest crown distance from the stem summed across all height classes represented the crown’s most prevalent growth direction.

Each ITC had a portion of crown in which the alphashapes from the 2 acquisition dates overlapped vertically (i.e., where a tree’s 2021-crown and 2017-crown 3D mesh overlapped; e.g., Fig. 3C and D). We calculated the differences in crown horizontal distances for the 36 available azimuths from the stem between years within this overlapping subset, and summed them for all overlapping height classes. The final 3 metrics were as follows:

3. We excluded any negative values (i.e. loss), and summed positive cumulative values across all heights, or largest growth (LG) 2D projection azimuth.
4. We calculate the azimuth with the largest negative value (excluding positive ones) or largest loss (LL) 2D projection azimuth.
5. Finally, we calculated the 2D projection azimuth with the largest absolute change (AC), i.e., positive or negative, from the vertically overlapping region.

In order to compare to our field estimates of crown within and between row-diameter measurements, we combined the largest individual distance values from (a) 50° and 230° and (b) 140° and 320° (regardless of height) for each tree to emulate a diameter measurement. Thus, we compared within and between row diameter between 2017 and 2021, in addition to both ITC delineation methods.

#### 3D shape complexity measures

As a measure of crown plasticity, we estimated crown shape complexity using the following complexity index: the OPCR. This index was calculated using the opcr function using the shape.index function, as implemented in the doolkit R package [74]. The OPCR was calculated from the 3D alphashape for all 2017 and 2021 ITCs.

#### Calculating 3D crown volumes in different azimuths

**Fig. 4.**
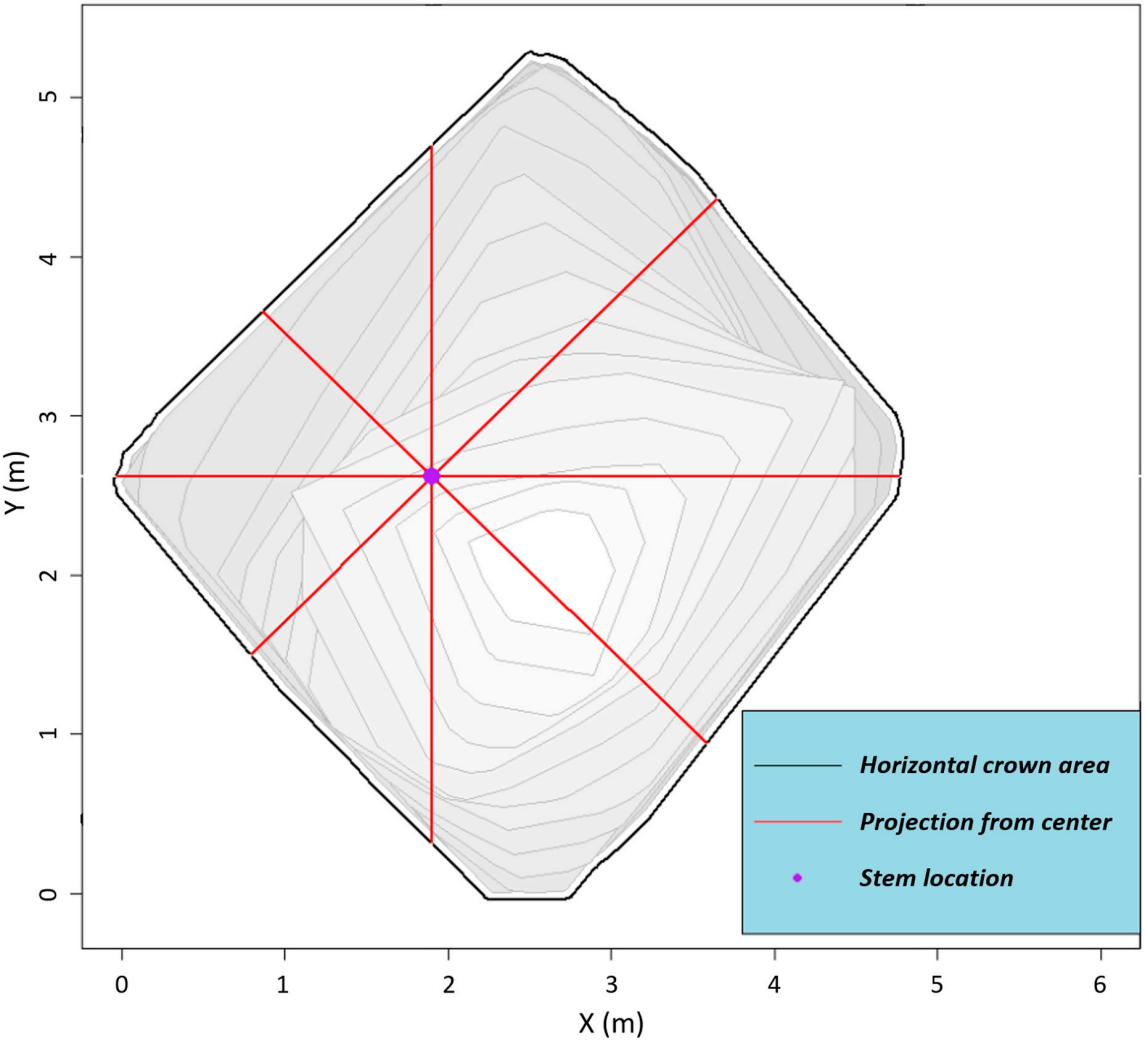
Wedge calculation example. The 3D mesh is subdivided into 45° segments horizontally around the stem center location. Each of the red lines represents a dividing plane. The gray stacked polygons represent horizontal cross-sections at different heights.

To evaluate crown volume changes in any potential direction between 2017 and 2021, we calculated the following for each of the ITC 3D alphashapes: A 3D voxel grid was created, where each cell was sized 0.1 × 0.1 m horizontally and 0.5 m vertically (0.1 × 0.1 × 0.5 m). We intersected this grid with the 3D mesh. Cells that did not intersect within the 3D mesh were removed. We created search areas, 3D wedges, originating from the tree stem. Each of these wedges covered a horizontal 45° span (i.e., 0° to 45°, 45° to 90° … 315 to 360°), resulting in 8 wedges per tree crown (Fig. 4). We then classified all voxels (via centroid coordinates) that intersected within each of the search wedges’ azimuth angles and summed their incident voxel volumes.

We also tested if there were any specific differences between the DLS-derived alphashape crown volume for the north- and south-facing crown segments per crown. We summed the volume of 2 wedges for north (315° to 0° and 0° to 45°) and 2 wedges for south (135° to 180° and 180° to 225°) and calculated the 3D mesh volume. This was computed for all crowns for 2017 and 2021.

### Statistical analysis

#### Circular analysis of potential crown foraging direction

We used the circular R package to calculate the metrics related to the largest projection azimuth for all trees. As described earlier, there were 5 foraging metrics per tree (as described in the “2D crown growth directionality” section). We assessed these metrics by (a) all stems, (b) the 6 genotypes, and (c) 4 blocks. We first calculated the circular mean of the ITCs, variance, and standard deviation (as in [75,76]). We also performed 2 statistical tests on all of our data by year (2017 and 2021). The first was the Rayleigh test of uniformity in order to determine if a dataset of azimuths was different from random (as in [75,76]) and was applied to all 5 azimuth metrics for both years. The second test, Watson’s 2-sample test of homogeneity, was performed between the largest 2D projection azimuth for 2017 and 2021 only. We used this to evaluate if the 2 groups, 2017 and 2021, had a different preferred foraging direction (as in [83 94]).

#### Evaluating differences in row direction on tree size

We examined the potential influence of the differences in our 2 plot layouts (blocks 1 to 3 and block 4) on both field-measured stem size (volume and DBH) and crown volume as derived from DLS analysis, and the difference in crown volume between the 2 dates. Mixed-effects model analyses were implemented using the lmer function in the lme4 package in R [77]. Either stem or crown size was set as the dependent variable, and both genotype and study block were fixed effects. The random effect included the unique plot identifier (*PlotID*) in each model. The models were assessed using a mixed-effects type III analysis of variance (ANOVA), with Satterthwaite’s method. Post hoc pairwise comparisons were made using the least squares means (lsmeans) function in the emmeans package [78].

#### Tree crown complexity mixed model analysis

We examined the influence of 3D alphashape mesh complexity (OPCR) and crown volume on the change of field-measured (a) stem volume and (b) DBH using both linear models and linear mixed-effects models as implemented in R. Potential outliers were detected using Cook’s distance and removed. We considered any values greater than 3 times the mean as outliers. Where appropriate, linear multiple regression models were evaluated with regard to multicollinearity using variance inflation factor (VIF). Any VIF values in excess of 2 were considered multicollinear, the variable was removed, and the model was rerun.

Mixed-effects model analyses were implemented, where either stem volume or DBH was our dependent variable. The two ITC metrics, (a) crown volume and (b) the OPCR index, were separately used as a fixed effect variable within the model. Each of these variables was fit using the lmer function in the lme4 package in R [77]. We evaluated various permutations of including genotype and study block as fixed effects. The unique plot identifier was included as the random effect in each model.

The model permutations were assessed using a mixed-effects type III analysis of covariance (ANCOVA), with Satterthwaite’s method. Significance values, *P*, were calculated using the lmerTest package [79]. Specifically, marginal and conditional correlation coefficients ($R_{m}^{2}$ and $\;R_{c}^{2}$, respectively) were computed using the MuMIn package [80]. Marginal *R*^2^ represents the variance explained by the fixed effects only, whereas the conditional *R*^2^ is interpreted as the variance explained by both fixed and random effects.

We also evaluated crown volume against the indices of complexity in order to determine if a larger crown simply means a greater shape complexity, rather than other factors. We evaluated the data from 2017 and 2021, separately. As before, we used both linear models and linear mixed-effects models. For the former, we explored if there was a correlation between stem volume or DBH change as respective dependent variables and the OCPR index and crown volume as the respective independent variables. For the latter, we implemented a linear mixed-effects analysis to test a new variable (*VI_n_*) calculated from the combination of crown volume and complexity index (i.e., *VI_n_* = *CVol_n_*/*OPCR_n_*), and if there was any interaction with genotype as a fixed effect for both years, and evaluated this value against stem volume change from 2017 to 2021 (*SVC*). We then evaluated the following mixed-effects model per year (*n*):

$$\mathrm{SVC}={\mathrm{VI}}_{n}+\mathrm{Gen}+\left(1|\text{PlotID}\right)$$ (4)

The model permutations were assessed using a mixed-effects type III ANCOVA, with Satterthwaite’s method. Pseudo-correlation coefficients for generalized mixed-effects models were calculated.

#### Tree crown volume in different azimuth mixed model analysis

We initially evaluated the impact of genotype on total individual crown volume change from 2017 to 2021 (C*Vol_change_*) as derived from DLS 3D alphashape, using mixed-effects model analysis:

$${\text{CVol}}_{\text{change}}=\mathrm{Gen}+\left(1|\text{PlotID}\right)$$ (5)

where *Gen* is the genotype and a fixed effect and *PlotID* is the unique plot identifier, included as the random effect.

We evaluated the impact of genotype on crown “wedge” volume growth for various azimuths (horizontal 45° segments). We then implemented mixed-effects model analysis, with wedge volume as our dependent variable. Wedge azimuth (×8), genotype, and study block were included as fixed effects. The unique plot identifier was included as a random effect. As before, the model permutations were assessed using a mixed-effects type III ANOVA, with Satterthwaite’s method.

We then used the computed crown volume for 2 directions, specifically north and south, derived from the DLS alphashape per crown and the difference between corresponding crown volume segments from 2017 and 2021 (90° north and south). We evaluated stem volume change from 2017 to 2021 (*SVC*), in addition to stem volume in 2017 (*SV_2017_*) and in 2021 (*SV_2021_*), as derived from the field data, as a dependent variable for mixed-effects modeling. Each of the various crown segment volumes (north and south for both 2017 and 2021) was used within the model as a fixed effect. The unique tree ID was included as the random effect. These models were assessed using mixed-effects type III ANCOVA, with Satterthwaite’s method.

Lastly, we evaluated if genotype interacted with the azimuth of the crown wedge’s volume and volume change. We implemented a mixed-effects model with repeated-measures analysis to evaluate stem volume change from 2017 to 2021, in addition to stem volume in 2017 and in 2021, as a dependent variable. The rows of the dataset represented individual crown segments. Crown segment volumes (2017, 2021, or volume change) were included in all models as a fixed effect. A variable for crown segment-facing direction was added (north or south) and included as a fixed effect in all models. Genotype was also included as a fixed effect in all models. As before, the model permutations were assessed using a mixed-effects type III ANCOVA, with Satterthwaite’s method.

We calculated the absolute change in crown volume per wedge (×8) using the following equation:

$${\mathrm{CV}}_{\text{wedge}}=\left|\frac{\left({\mathrm{Vol}}_{2021}-{\mathrm{Vol}}_{2017}\right)}{{\mathrm{Vol}}_{2017}}\right|\times 100$$ (6)

where *CV_wedge_* is the absolute change in wedge volume for a single 45° wedge, and *Vol_2017_* and *Vol_2021_* are the volume (m^3^) of the single crown wedge derived from 2017 and 2021 DLS data, respectively. Average volume change per wedge per genotype was calculated for visual comparison.

## Results

### Tree growth summary and ITC accuracy

Field measurements recorded 1,728 field stems in 2017 and 1,695 field stems in 2021. The difference in stem counts was due to tree mortality. A summary of stem volume in 2017 and annual increase is presented in Table 3. The first ITC delineation procedure (as in [38]) correctly located 98 and 99% of stems for 2017 and 2021, respectively. The PTree method correctly located 95 and 98% stems for 2017 and 2021, respectively. Between 0 and 6 additional ITC objects were erroneously delineated using the PTree method per plot.

**Table 3.** Summary of mean and total stem volume change by genotype (*n* = 6) and block (*n* = 4)

Data	Stem volume	
	Mean in 2017 (m^3^)	Annual growth rate (m^3^)
Genotype 1	0.17	0.08
Genotype 2	0.15	0.06
Genotype 3	0.15	0.07
Genotype 4	0.17	0.07
Genotype 5	0.14	0.07
Genotype 6	0.15	0.07
Block 1	0.17	0.08
Block 2	0.16	0.07
Block 3	0.15	0.07
Block 4	0.14	0.07

We evaluated accuracy of the estimated stem position horizontal coordinates against field-measured planting GPS coordinates. The predicted stem locations from the Sumnall et al. [38] ITC method has a mean horizontal difference of 0.49 m (standard deviation, 0.15 m) between GPS and DLS position. For PTree, the mean horizontal difference was 0.50 m (standard deviation, 0.16 m). The GPS position was consistently northwest of the estimated stem locations, indicating systematic error.

We compared crown field diameter measurements against the largest individual distance values derived from the ITC alphashape distances for (a) 50° and 230° and (b) 140° and 320° (regardless of height). The results of the 2 ITC methods are summarized in Table 4. The results by Sumnall et al. [38] showed a slightly higher overall accuracy (<0.4 m) than PTree; thus, we selected the outputs of the former for subsequent analysis.

**Table 4.** Summary of the crown diameter estimates derived from 2 drone laser scanning individual tree crown (ITC) delineation methods: (a) Sumnall et al. [38] and (b) PTree [68]. Absolute and normalized root mean square error (RMSE and NRMSE, respectively) and absolute and normalized bias (Bias and NBias, respectively) were computed considering the difference to field measurements of crown diameter in 2017 and 2021. Two measurements of crown diameter were compared: (a) those in line with, or within, the planting row and (b) perpendicular to the planting row or between row.

ITC method	Year	Crown diameter type	RMSE (m)	NRMSE (%)	Bias (m)	NBias (%)
Sumnall	2017	Within row	0.74	15	−0.14	−3
	2017	Between row	0.76	18	0.05	1
	2021	Within row	1.19	15	−0.49	−6
	2021	Between row	1.17	17	−0.36	−5
PTree	2017	Within row	1.05	22	−0.23	−5
	2017	Between row	1.10	21	−0.42	−8
	2021	Within row	1.48	22	−0.53	−8
	2021	Between row	1.57	20	−0.74	−9

### Circular analysis of potential crown foraging direction

We evaluated the largest cumulative horizontal projection direction for all trees, which are summarized in Table 5. The results indicated a similarity in mean azimuth for 2017 and 2021 (152° and 160°, respectively, or ±8°). The Rayleigh test of uniformity was significant (*P* < 0.05), suggesting that individuals are larger in a preferred direction and not at random. The Watson’s 2-sample test of homogeneity of largest mean azimuth of 2017 against 2021 was not significant (*P* > 0.05), suggesting that the 2 groups have the same preferred direction. The Rayleigh test also indicated that there was a significant directionality to the azimuth of the LL (mean of 10.2°).

**Table 5.** Summary of circular statistics for all ITCs of the most prevalent growth direction. Results are presented for the azimuth with the largest values for the years 2017 and 2021, the azimuth of the largest growth (LG), largest loss (LL), and absolute change (AC). The symbol “*” denotes a significant test result (*P* < 0.05).

Data	Circular mean (°)	Variance (°)	Standard deviation (°)	Rayleigh *P*
2017	151.97	47.59	107.96	0.00*
2021	159.79	41.69	92.41	0.00*
LG	199.10	55.20	147.40	0.17
LL	10.20	54.54	141.14	0.05*
AC	281.97	56.06	2.77	0.54

When stratifying the results by genotype, summarized in Table 6, the circular mean for 2017 varied from 101 to 188° between genotypes, whereas in 2021 the circular mean varied from 150 to 173° between genotypes. There was a difference between the mean largest azimuths in 2017 and 2021 for each genotype. The largest difference was observed for genotype 2 (46°). Circular variance and standard deviation of the largest projection azimuth could vary by ±16.1° and ±49.5° (Table 6), respectively. When evaluated with the Rayleigh test, with the exception of genotypes 2 and 6, all data from 2017 were significant, indicating non-random directionality to growth. All Rayleigh test results from 2021 were significant. The Watson’s 2-sample test was significant only for genotypes 2 and 6, indicating different preferred directions between the 2 dates.

**Table 6.** Summary of circular statistics for ITCs, stratified by genotype (G1 to G6), of the most prevalent growth direction. Results are presented for the azimuth with the largest values for the years 2017 and 2021, the azimuth of LG, LL, and AC. The symbol “*” denotes a significant test result (*P* < 0.05).

Genotype	Data	Circular mean (°)	Variance (°)	Standard deviation (°)	Rayleigh *P*
G1	2017	156.01	48.41	110.61	0.01*
	2021	149.81	40.57	89.92	0.00*
	LG	186.48	51.81	124.09	0.15
	LL	340.67	52.71	128.76	0.27
	AC	127.29	56.86	178.97	0.99
G2	2017	101.92	51.51	122.68	0.11
	2021	157.13	37.34	83.21	0.00*
	LG	170.43	45.83	102.79	0.00*
	LL	10.63	49.22	113.43	0.01*
	AC	171.73	51.42	122.30	0.10*
G3	2017	146.24	42.86	95.15	0.00*
	2021	173.15	43.84	97.53	0.00*
	LG	252.40	53.42	132.98	0.35
	LL	64.19	48.14	109.73	0.00*
	AC	263.16	54.56	141.33	0.59
G4	2017	155.84	40.39	89.51	0.00*
	2021	165.35	41.06	90.98	0.00*
	LG	301.11	50.05	116.52	0.02
	LL	168.02	53.55	133.80	0.36
	AC	300.02	53.31	132.31	0.32
G5	2017	162.13	46.86	105.74	0.00*
	2021	151.43	45.18	100.99	0.00*
	LG	1.83	52.03	125.21	0.16
	LL	257.65	52.43	127.22	0.21
	AC	322.08	50.18	117.02	0.04
G6	2017	188.91	53.43	133.06	0.38
	2021	161.40	41.22	91.35	0.00*
	LG	125.45	50.94	120.15	0.07
	LL	340.64	51.55	122.87	0.12
	AC	85.09	54.77	143.20	0.66

To reiterate, the following LG, LL, and AC metrics were computed for the overlapping portions of the ITC alphashape meshes. The mean LG azimuth varied between genotypes. For genotypes 4 and 5, this was 302° and 2°, respectively; for genotypes 1 to 3, this was 125° to 186°; and finally genotype 3 was 252°. Except for genotype 2, the Rayleigh test was insignificant, meaning that the LG for all trees was mostly random. Mean LL was generally in the opposite direction to LG, apart from genotype 5. LL ranged from 340° to 341° for genotypes 1 and 6, and 10° to 64° for genotypes 2 and 3. For genotype 4, LL was 168°, and for genotype 5, LL was 257°. Except for genotypes 2 and 3, the Rayleigh tests were insignificant. The AC was very similar to LG for genotypes 2 to 4 (<10° difference). The Rayleigh tests were insignificant for AC, with the exception of genotype 2.

When stratifying the results by treatment block to investigate if differences in row-direction influences crown growth—summarized in Table 7—the circular means of largest projection for 2017 and 2021 for block 4 were noticeably different than the others (±67° and ±38°, respectively). All Rayleigh test data from 2017 and 2021 largest projections azimuths were significant, implying a preferred direction. The Watson test, comparing 2017 to 2021 largest projection azimuth, was significant for blocks 1 to 3. Among all tests, crown projection tended to be greater in a southerly direction and projection losses tended to be in a non-southerly direction. Specifically, 2021 mean largest projection azimuth varied from −16° to −34° from true south (i.e., |*x* − 180|) for blocks 1 to 3, and +5° for block 4. The LL varied from 85° to 176° for blocks 1 to 3, relative to true south, and 84° for block 4. The analysis by block indicates that while growth still tends to be southerly, the crowns will grow where space is available in a southerly direction.

**Table 7.** Summary of circular statistics for ITCs, stratified by site block (1 to 4), of the most prevalent growth direction. Results are presented for the azimuth with the largest values for the years 2017 and 2021, the azimuth of LG, LL, and AC. The symbol “*” denotes a significant test result (*P* < 0.05).

Block number	Data	Circular mean (°)	Variance (°)	Standard deviation (°)	Rayleigh *P*
Block 1	2017	139.30	42.08	93.30	0.00*
	2021	159.80	40.10	88.88	0.00*
	LG	317.58	52.71	128.75	0.17
	LL	94.19	53.58	134.04	0.31
	AC	298.98	54.21	138.53	0.45
Block 2	2017	136.34	48.44	110.72	0.00*
	2021	146.22	38.45	85.44	0.00*
	LG	174.60	53.61	134.21	0.21
	LL	355.59	53.13	131.22	0.14
	AC	208.24	55.73	153.72	0.76
Block 3	2017	154.59	44.86	100.16	0.00*
	2021	163.94	37.91	84.35	0.00*
	LG	186.58	49.36	113.93	0.00*
	LL	27.91	49.61	114.84	0.00*
	AC	299.75	56.40	165.16	0.92
Block 4	2017	203.04	50.98	120.31	0.02*
	2021	184.85	49.00	112.64	0.00*
	LG	17.31	55.92	156.46	0.82
	LL	264.04	52.86	129.58	0.13
	AC	312.82	56.04	158.35	0.85

### Evaluating differences in row direction on tree size

For field-measured stem volume and DBH, from both 2017 and 2021, as the dependent variables in mixed-effects analysis, block, genotype, and the interaction between the 2 were not significant. The post hoc pairwise comparison indicated that trees in block 4 (spaced widest along a 45° azimuth) were smaller than trees in the other 3 blocks (spaced widest along a 135° azimuth).

For both dates (2017 and 2021), block and genotype were significant as main effects (*P* = 0.01) in predicting crown volume. For crown volume change, both block and genotype were, again, significant (*P* < 0.01). The interaction between block and genotype was not significant in all models. Post hoc tests indicated that trees in 2017 block 4 (spaced widest along a 45° azimuth) had crown volumes that were typically smaller than trees in the other 3 blocks (spaced widest along a 135° azimuth), but the opposite was true in 2021. Crown volume change between 2017 and 2021 was smaller in blocks 1 to 3. Crown volume change in block 1 to 3 was 59.8 m^3^ tree^−1^ [±1.35, 99% confidence interval (CI) = 55.7:63.9 m^3^ tree^−1^]. Crown volume change in block 4 was 71.9 m^3^ tree^−1^ (±2.33, 99% CI = 64.9:79.0 m^3^ tree^−1^).

### Tree crown complexity and volume mixed model analysis

#### Assessment of crown variables and stem volume change

**Fig. 5.**
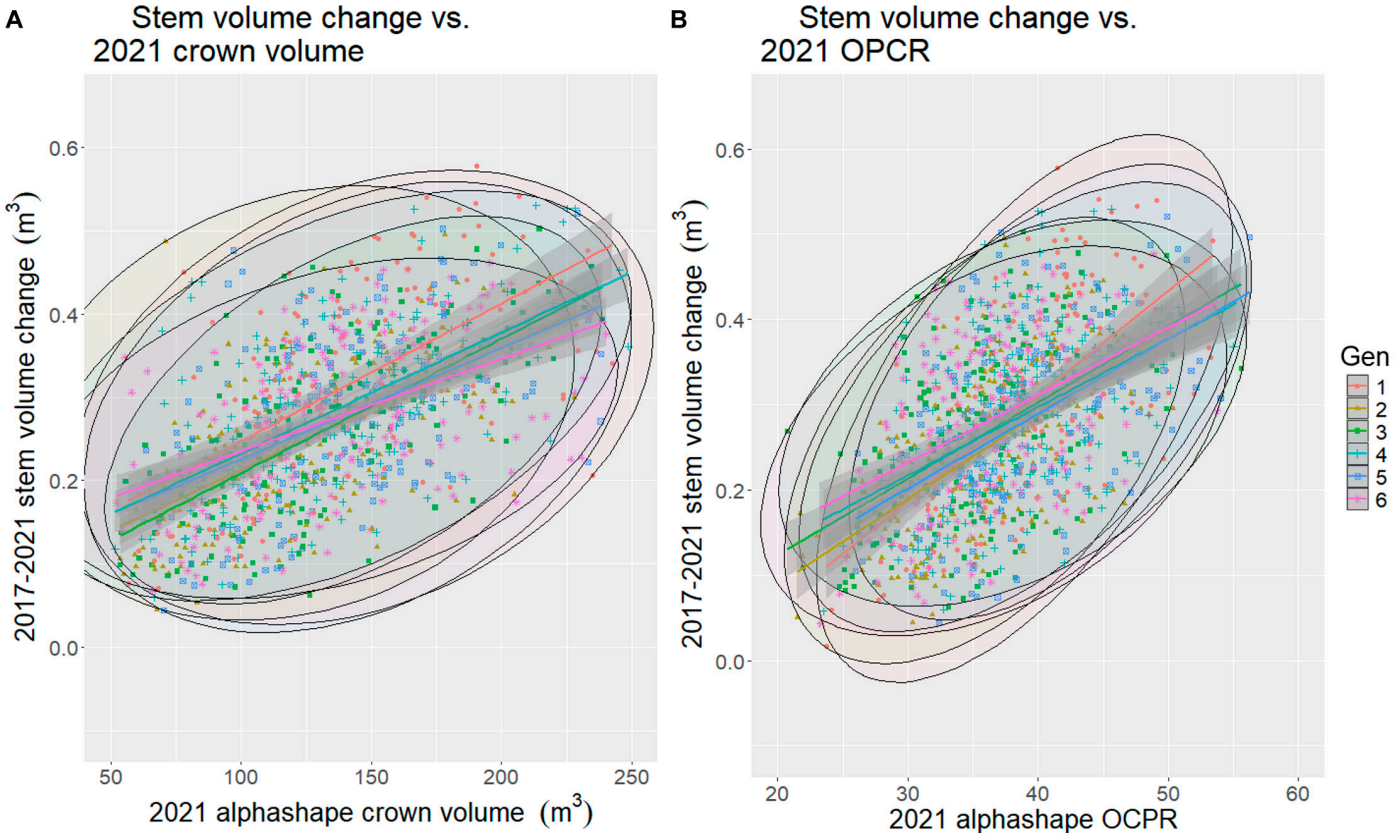
Scatterplots generated from the mixed-effects model generated for 2017 to 2021 stem volume change against either (A) 2021 alphashape crown volume or (B) 2021 alphashape orientation patch count rotated (OPCR) index and loblolly pine genotype (1 to 6).

Each of the models presented in Table 8 had significant interactions when predicting stem volume change (*P* < 0.05). The highest marginal *R*^2^ values were observed for models using crown volume and OPCR (*R*^2^ = 0.24:0.34). The 2 respective best-performing models, in terms of pseudo-*R*^2^ value, are illustrated in the scatterplots of Fig. 5, where larger crown volume, and complexity, correlated with higher stem volume change; however, difference in genotypes was present.

**Table 8.** Summary of significant (*P* < 0.05) only mixed-effects models predicting stem volume change between 2017 and 2021 using crown metrics and genotype. Marginal ($R_{m}^{2}$) and conditional ($R_{c}^{2}$) pseudo-*R*^2^ metrics are reported and represent the variance explained by the fixed effects and the entire model, respectively. The terms are defined as follows: *SVC* is the change in stem volume from 2017 and 2021, *OPCR_yr_* is the orientation patch count rotated index ([56–58]), and *CVol_yr_* denotes crown volume, where *yr* can represent data from the year 2017 or 2021. Random effects included the unique plot identifier (*PlotID*).

Variable	Significant mixed-effects model equation	$R_{m}^{2}$	$R_{c}^{2}$
OPCR	*SVC* = *OPCR*_2017_ × *Gen* + (1| *PlotID*)	0.24	0.31
OPCR	*SVC* = *OPCR*_2021_ × *Gen* + (1| *PlotID*)	0.24	0.29
OPCR	*SVC* = (*OPCR*_2021_ − *OPCR*_2017_) × *Gen* + (1| *PlotID*)	0.24	0.29
CVol	*SVC* = *CVol*_2017_ × *Gen* + (1| *PlotID*)	0.34	0.40
CVol	*SVC* = *CVol*_2021_ × *Gen* + (1| *PlotID*)	0.34	0.41
CVol	*SVC* = (*CVol*_2021_ − *CVol*_2017_) × *Gen* + (1| *PlotID*)	0.34	0.41

In contrast to stem volume change, more of the complexity indices were significantly related to DBH change (*P* < 0.05; Table 9). The highest marginal *R*^2^ values were observed for models using crown volume (Table 9).

**Table 9.** Summary of significant (*P* < 0.05) only mixed-effects models predicting diameter at breast height (DBH) change between 2017 and 2021 using crown metrics and genotype. Marginal ($R_{m}^{2}$) and conditional ($R_{c}^{2}$) pseudo-*R*^2^ metrics are reported and represent the variance explained by the fixed effects and the entire model, respectively. The terms are defined as follows: *DBH_change_* is the change in DBH from 2017 and 2021, *OPCR_yr_* is the orientation patch count rotated index [56–58], and *CVol_yr_* denotes crown volume, where *yr* can represent data from the year 2017 or 2021. The unique plot identifier (*PlotID*) is the random effect.

Variable	Significant mixed-effects model equation	$R_{m}^{2}$	$R_{c}^{2}$
OPCR	*DBH_change_* = *OPCR*_2017_ × *Gen* + (1| *PlotID*)	0.17	0.34
CVol	*DBH_change_* = *CVol*_2017_ × *Gen* + (1| *PlotID*)	0.19	0.33
CVol	*DBH_change_* = *CVol*_2021_ × *Gen* + (1| *PlotID*)	0.28	0.37
CVol	*DBH_change_* = (*CVol*_2021_ − *CVol*_2017_) × *Gen* + (1| *PlotID*)	0.28	0.37

We further explored those variables that significantly predicted stem volume or DBH change, and which maximized pseudo-*R*^2^, by generating linear model coefficients and assessing if there were differences between genotypes. These models included OPCR and crown volume as covariates (in Table 10). Each of the linear model coefficients were similar or identical (<0.02) for each of the 6 genotypes. For stem volume change, intercept values varied from −0.08 to 0.03 and −0.13 to 0.01 for *CVol*_2017_ and *OPCR*_2017_, respectively, across the genotypes. For DBH change, intercept values varied from 0.22 to 1.77 for the *CVol*_2021_ metric for each genotype.

**Table 10.** Summary of linear models generated from a subset of mixed-effects models that were significant (*P* < 0.05) and that maximized pseudo-*R*^2^. Linear model intercepts generated for the crown complexity/volume metric for each of the genotype factors included. *OPCR_yr_* [56–58] is the orientation patch count rotated index and *CVol_yr_* denotes crown volume, where *yr* can represent data from the year 2017 or 2021.

Dependent variable	Independent variable		Genotype					
			G1	G2	G3	G4	G5	G6
*V_change_*	*CVol*_2017_	Intercept	−0.03	−0.01	−0.09	−0.09	−0.00	0.03
		Coefficient	0.01	0.00	0.01	0.00	0.00	0.00
		*R* ^2^	0.37	0.23	0.41	0.36	0.24	0.24
*V_change_*	*OPCR*_2017_	Intercept	−0.13	0.01	−0.08	−0.09	−0.12	0.02
		Coefficient	0.02	0.01	0.01	0.01	0.01	0.01
		*R* ^2^	0.24	0.09	0.22	0.21	0.21	0.13
*DBH_change_*	*CVol*_2021_	Intercept	1.40	1.22	0.22	0.84	1.29	1.77
		Coefficient	0.01	0.01	0.02	0.01	0.01	0.01
		*R* ^2^	0.25	0.15	0.39	0.30	0.22	0.14

#### Evaluation of alphashape volume and complexity

We evaluated the relationship between the OPCR and DBH using linear regression (Table 11). All 3 models were significant for *OCPR_2017_*, *OPCR_2021_*, and combined (*OCPR_2017_* and *OPCR_2021_*) and produced *R*^2^ values ranging from 0.08 to 0.16. When the dependent variable was changed to stem volume, *R*^2^ values ranged from 0.19 to 0.29. All VIF values were below 1.65. Models including metrics related to crown volume (for 2017 or 2021) generally had the smallest Akaike information criterion (AIC) value, particularly for stem volume change (*SVC*). For DBH change (*DBH_change_*), the smallest AIC values were observed for models including OCPR and crown volume metrics from 2021.

**Table 11.** Summary of linear regression model coefficients, where the crown complexity index orientation patch count rotated (*OPCR*) computed from an alphashape fit to an individual tree classified DLS point cloud acquired in either 2017 or 2021 is regressed against field-measured stem size change metrics: stem volume or diameter at breast heights. Change (*SVC* and *DBH_change_*) was calculated as the difference between measurements in 2017 and 2021. Additional models were created, which include both *OPCR_yr_* and crown volume (*CVol_yr_*), where *yr* denotes that the metric is calculated from 2017 or 2021 data. Akaike information criterion (AIC) values are also reported.

Dependent variable	Independent variable(s)	*R* ^2^	Intercept	Coefficient(s)	AIC
*SVC*	*CVol_2017_*	0.27	0.01	0.003	−2,386.20
*SVC*	*CVol_2021_*	0.31	−0.04	0.002	−2,457.60
*SVC*	*CVol_2017_*	0.37	−0.10	0.002	−2,570.44
	*+ CVol_2021_*			0.002	
*SVC*	*OCPR_2017_*	0.19	−0.06	0.011	−2,261.51
*SVC*	*OCPR_2021_*	0.22	−0.11	0.010	−2,300.58
*SVC*	*OCPR_2017_*	0.29	−2.38	0.008	−2,419.28
	+ *OPCR_2021_*			0.008	
*SVC*	*OCPR_2017_*	0.29	−0.08	0.003	−2,420.49
	*+ CVol_2017_*			0.005	
*SVC*	*OPCR_2021_*	0.34	−0.15	0.002	−2,511.58
	*+ CVol_2021_*			0.005	
*SVC*	*OCPR_2017_*	0.40	−0.22	0.002	−2,626.51
	*+ CVol_2017_*			0.002	
	*+ OPCR_2021_*			0.004	
	*+ CVol_2021_*			0.001	
*DBH_change_*	*CVol_2017_*	0.09	1.92	0.013	2,602.00
*DBH_change_*	*CVol_2021_*	0.25	1.05	0.014	2,358.49
*DBH_change_*	*CVol_2017_*	0.25	1.01	0.001	2,359.77
	*+ CVol_2021_*			0.013	
*DBH_change_*	*OCPR_2017_*	0.08	1.49	0.050	2,613.55
*DBH_change_*	*OCPR_2021_*	0.14	0.84	0.056	2,531.71
*DBH_change_*	*OCPR_2017_*	0.16	0.38	0.028	2,502.85
	+ *OPCR_2021_*			0.046	
*DBH_change_*	*OCPR_2017_*	0.11	1.43	0.009	2,582.20
	*+ CVol_2017_*			0.028	
*DBH_change_*	*OPCR_2021_*	0.27	0.60	0.012	2,342.63
	*+ CVol_2021_*			0.019	
*DBH_change_*	*OCPR_2017_*	0.27	0.45	0.010	2,340.59
	*+ OPCR_2021_*			0.017	
	*+ CVol_2021_*			0.001	

We evaluated any potential relationship between alphashape-derived crown volume and shape complexity, initially using linear regression. Regressing *OPCR_2017_* against *CVol_2017_* (dependent variable) produced an *R*^2^ value of 0.39 (*P* < 0.05) and model coefficients of 6.75 for the intercept and 2.44 for *OPCR_2017_*. The model concerning 2021 data, where *CVol_2017_* is the dependent variable, produced an *R*^2^ value of 0.34 (*P* < 0.05), with the following coefficients: 18.83 for the intercept and 3.22 for *OPCR_2021_*.

When *OPCR_yr_* and *CVol_yr_* (where *yr* is the year 2017 or 2021) were included in the same model and regressed against stem volume change (as the dependent variable), all inputs were significant (*P* < 0.05) and produced *R*^2^ values of 0.29 and 0.34 for 2017 and 2021, respectively (Table 11). When all 4 metrics (*OPCR_2017_*, *OPCR_2021_*, *CVol_2017_*, and *CVol_2021_*) were used in the same model, all variables were significant (*P* < 0.05) and *R*^2^ increased to 0.4. We then regressed both *OPCR_yr_* and *CVol_yr_* against DBH change, and again all model inputs were significant (*P* < 0.05). The models produced *R*^2^ values of 0.11 and 0.26 for 2017 and 2021, respectively. When all 4 metrics were combined in a single model, only *CVol_2017_* was insignificant (*P* > 0.05). When this variable was removed, *R*^2^ was 0.27. All VIF values for all models were below 1.9.

Both *VI_2017_* (β_1_ = 0.068) and *VI_2021_* (β_1_ = 0.56) (a year’s crown volume divided by its corresponding complexity index value) were significantly (*P* < 0.05) related to stem volume change, although liner model *R*^2^ values were low (0.08 and 0.09). In the mixed-effects model analysis, block did not significantly interact with *VI* values. Interactions between *VI_2017_* and genotype were significant (*P* < 0.05), with marginal and conditional pseudo-*R*^2^ metrics being 0.13 and 0.20, respectively. The model including *VI_2021_* was not significantly (*P* > 0.05) related to stem volume change.

### Tree crown volume in different azimuth mixed model analysis

In a mixed-effects model (Eq. 5), crown volume change was significantly related to genotype (*P* < 0.05), where marginal and conditional pseudo-*R*^2^ metrics were 0.08 and 0.19, respectively.

In a mixed-effects model, the main effects and interaction of genotype and wedge azimuth were significant (*P* < 0.05) in predicting wedge volume change (×8; derived from portions of the DLS alphashape). Post hoc test results are presented in Table 12. Of the 120 comparisons, 56 were significant, suggesting that genotypes have unique crown morphologies. There were 2 contrasts between genotypes G1 to G5 and G2 to G6, where no significant differences were observed for all directions. G1 and G5 tended to grow more volume than others among wedges, and G4 wedges were consistently smaller than other genotypes. G4 tended to allocate less crown volume in wedges between 90° and 270° than other genotypes.

**Table 12.** Significance values from the linear mixed-effects model pairwise contrasts for each of the genotype and azimuths. The center azimuths of each volume wedge were 22.5°, 67.5°, 112.5°, 157.5°, 202.5°, 247.5°, 292.5°, and 337.5°.The 6 genotypes are specified as G1, G2, G3, G4, G5, and G6. Pairwise comparisons (*P* < 0.05) are presented. A “>” symbol denotes that the left-most genotype (e.g., G1) was larger, and a “<” symbol indicates that the right-most genotype was a larger volume. Blank cells indicate that no significant differences exist.

Contrast			*P* value of center azimuth							
			22.5°	67.5°	112.5°	157.5°	202.5°	247.5°	292.5°	337.5°
G1	-	G2	>	>					>	>
G1	-	G3	>	>	>	>	>			
G1	-	G4	>	>	>	>	>	>		
G1	-	G5								
G1	-	G6	>	>					>	>
G2	-	G3				>			<	<
G2	-	G4	<		>	>	>			<
G2	-	G5	<	<					<	<
G2	-	G6								
G3	-	G4				>	>	>	>	
G3	-	G5	<	<	<	<				
G3	-	G6				<			>	>
G4	-	G5	<	<	<	<	<	<	<	<
G4	-	G6			<	<	<			
G5	-	G6	>	>	>				>	>

**Fig. 6.**
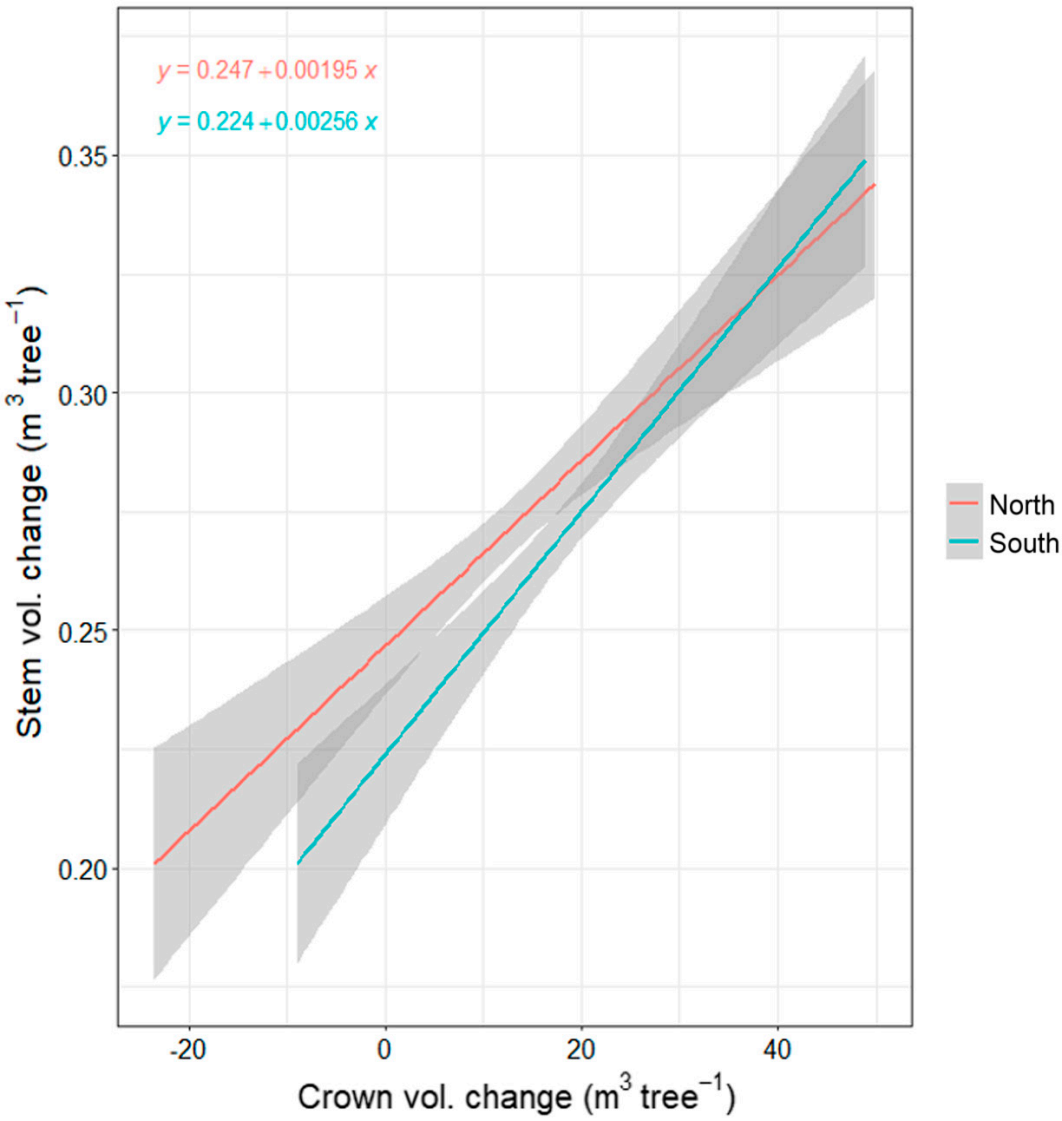
Individual tree stem volume change (2017 to 2021) was regressed against estimated 3D crown 90° segment volume oriented either north or south.

We computed the change in crown volume for 2 directions, north (*CVC_north_*) and south (*CVC_south_*), illustrated in Fig. 6. The efficiency of crown volume being converted to stem volume was greater when crown volume was added to the south than north. Greater negative crown volume change was observed in the north, as well. We used mixed-effects models to evaluate the direction of crown growth on stem volume change (*SVC_north_; SVC_south_; SVC_both_*). The fixed effects and interaction in each of the following 3 models were significant (*P* < 0.05):

$${\mathrm{SVC}}_{\text{north}}={\mathrm{CVC}}_{\text{north}}+\left(1|\text{treeID}\right)$$ (7)

$${\mathrm{SVC}}_{\text{south}}={\mathrm{CVC}}_{\text{south}}+\left(1|\text{treeID}\right)$$ (8)

$${\mathrm{SVC}}_{\text{both}}={\mathrm{CVC}}_{\text{north}}+{\mathrm{CVC}}_{\text{south}}+\left(1|\text{treeID}\right)$$ (9)

Marginal *R*^2^ values were 0.03, 0.04, and 0.09, with conditional *R*^2^ values of 0.12, 0.13, and 0.18, respectively, for Eqs. 7 to 9. Each of the 4 crown volumes (north and south for 2017 and 2021) were significantly related to stem volume change (*P* < 0.05). Marginal *R*^2^ values for northern segments ranged from 0.08 to 0.09, and conditional *R*^2^ values ranged from 0.16 to 0.17. Marginal *R*^2^ values for southern segments ranged from 0.11 to 0.15, and conditional *R*^2^ values ranged from 0.18 to 0.22.

The static estimates of north and south total crown volumes for both 2017 and 2021 significantly interacted with both direction and genotype when predicting 2017 stem volume and 2021 stem volume. No other interactions were significant. When predicting stem volume change, however, crown volume for either year did not interact with direction.

We evaluated static stem volume for both 2017 and 2021, and stem volume change using directional crown volume metrics. For the model predicting the change in stem volume between 2017 and 2021, the wedge crown volume change and direction were significant (*P* < 0.05; Fig. 6). Genotype and all interactions were not significant. Models predicting the change in stem volume, with crown wedge volume for either 2017 or 2021 and direction as main effects, were significant (*P* < 0.05). Genotype was significant, but the interactions were not significant.

**Fig. 7.**
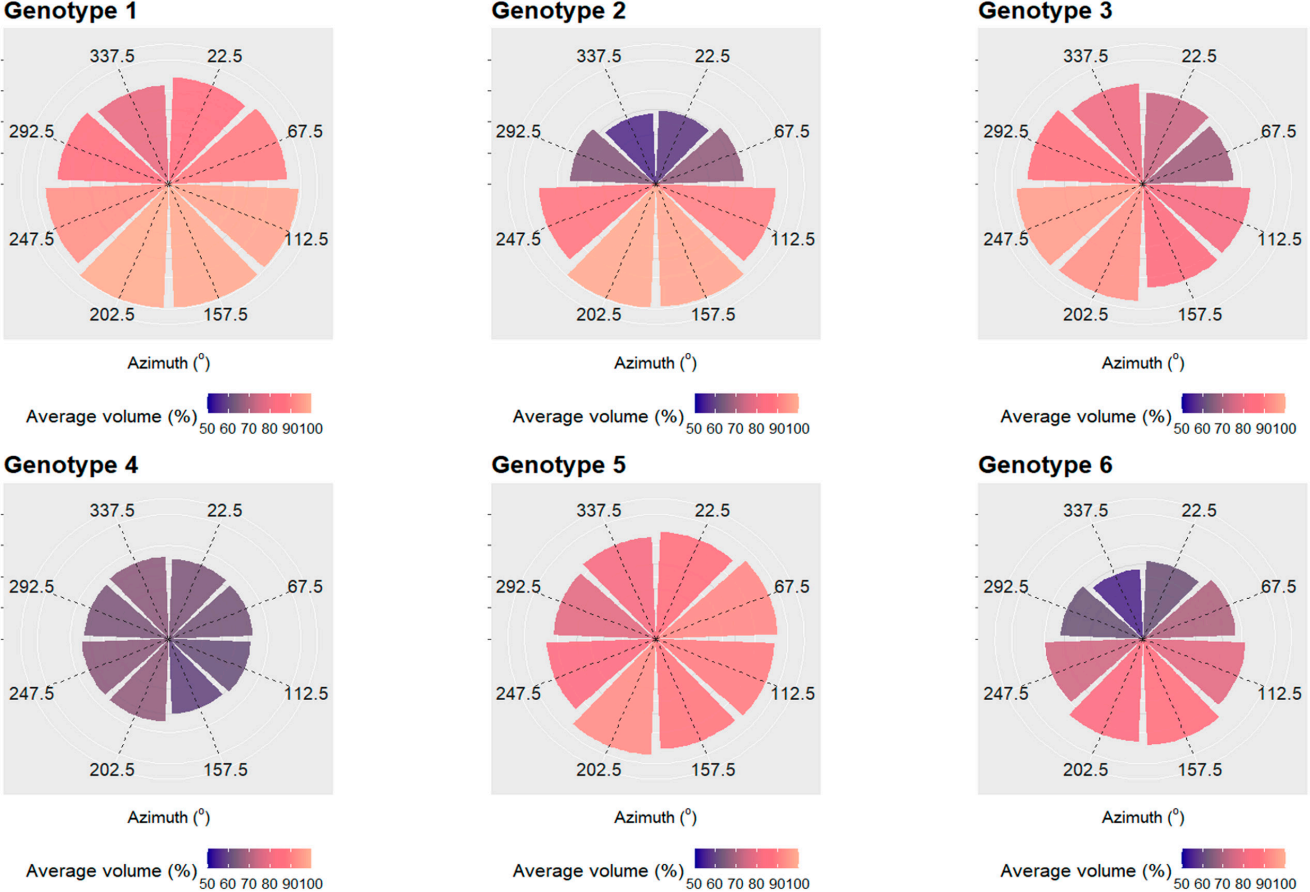
Each wedge (for 45° segments) represents the average difference in crown volume between 2017 and 2021 for each genotype. The wedges are orientated in their corresponding compass directions (0° to 360°) relative to the tree crown center. Volume is expressed as a percentage proportion increase of the crown volume as it was in 2017; thus, a value of 50% would indicate the 2017 volume plus 50%.

We calculated the average change in crown volume per 45° wedge per genotype for visual comparison (see Fig. 7). Average crown growth among wedges is variable across genotypes, with genotypes 1, 2, 3, and 6 exhibiting larger changes in southerly azimuths than genotypes 4 and 5. Genotypes 2 and 6 exhibit little change in northerly azimuths and larger changes in southerly azimuths. For genotype 5, this appears to be southeasterly. Genotype 4 shows the smallest change in overall crown volume.

## Discussion

The plasticity of crown morphologies varied significantly spatially, temporally, and among 6 genotypes of loblolly pine. We found that crown asymmetries are influenced by (a) the prevalent direction of solar radiation, (b) the spatial arrangement and proximity of the neighboring crowns (i.e., growing space), and (c) loblolly pine genotype. Our index of complexity was a weaker predictor of stem volume growth when compared to 3D alphashape crown volume, which added little predictive power when included in models predicting stem size along with crown volume. Growth efficiency—that is, stem volume added per unit of crown volume added—was greater for southern portions of crowns, in contrast to the northern portion. This finding, in addition to our analysis contrasting genotypes, suggests that row orientation could influence stem growth rates in plantations.

### Assessment of crown horizontal foraging direction

Trees consistently grew in a more southerly direction, and the southerly crown was more efficient at converting crown volume to stem volume. The ability to allocate crown volume also differed among the genotypes.

With the exception of the ITCs from genotypes 2 and 6 in 2017, all Rayleigh tests were significant (*P* < 0.05), suggesting that the ITCs have a preferred direction of growth across all genotypes and blocks. The overall average azimuth was 152° for 2017 and 160° for 2021. The majority of the direct radiation is from southern directions in this geographical region [33], which aligned with the largest volume of crown facing south.

There were differences between 2017 and 2021 directions for each of the genotypes, ranging between 6° and 55° when comparing means. When compared using the Watson test, genotypes 2 and 6 exhibited statistically significant different directions between the 2 acquisitions’ ITC populations. This suggests a potential for change in crown shape over time.

We calculated the change in direction for overlapping vertical regions of the 3D alphashapes. We should note that, within our study, the vertical overlap between the 2 times, 3D alphashapes for our matched ITCs was often limited, accounting for only small portions of the total crown. This could explain the increased variability when assessing azimuth of crown growth, loss, and absolute change. Genotypes 2 and 4 exhibited a significant azimuth for growth. Genotypes 2 and 3 exhibited a significant azimuth for loss. Only genotype 5 (control pollinated) had a significant absolute change. Genotype 6 (open-pollinated) met our expectation as being the most variable in terms of shape (as in [81]). The results relating to genotype 2 imply that it may have a tighter control over its growth directionality in contrast to the other genotypes tested.

The results reported here are supported by Chmura et al. [71], which evaluated 2 genotypes of loblolly pine, which each exhibited different crown and needle traits, and biomass partitioning patterns. Uria-Diez and Pommerening [82] assessed how Scots pine (*Pinus sylvestris*) individuals responded to the presence of neighbors and stated that trees were able to make an efficient use of the canopy space by shifting their crowns to reduce the inter-tree competition. As indicated in our results, crown shape changes over time. Uria-Diez and Pommerening [82] went on to state that young stands did not show crown displacements as much as mature stands. The results presented in Table 6 and Fig. 7 imply that crown asymmetry would increase with time for genotypes 2 and 6. Repeated DLS acquisitions may therefore allow us to monitor such changes through time.

There were differences observed in tree sizes between block 4 (spaced widest along a 45° azimuth) and the other 3 blocks (1 to 3; spaced widest along a 135° azimuth). The row spacing was 4.4 m within row and 3.7 m between rows; therefore, there was greater available space in the direction of the row. The different row orientation present in block 4 would also account for the different observed mean projection azimuth there. These row orientations would likely account for the difference between our observed mean values and true south. We assume that the only site differences were row orientation as the plots were equally intensively managed as a research trail. Block 4 crown volume was typically smaller than those of the other blocks in 2017 and larger in 2021. This may reflect that available crown growing space diminished for the more rapidly growing trees in blocks 1 to 3 by 2021, but crown growing space had yet to be fully occupied in block 4. This finding suggests that thinning may need to be conducted later in stands orientated at 45° and earlier in 135° as they occupy growing space faster and experience a reduced stem growth rate once horizontal crown growing space is fully utilized. Thus, for the former, growth is slowing down from 2017 to 2021.

These results contrast those of Amateis et al. [29], who stated that row azimuth had no significant impact on either basal area or height growth (over 20 years of observations). While there are few studies investigating the effects of row orientation on pine plantation productivity, there are several studies that have evaluated row orientation on crop or vineyard development. Hunter et al. [26] and Buesa et al. [27] noted differences in canopy light interception, photosynthesis, and water-use efficiency for disparate row orientations, but this did not significantly affect carbon assimilation. Others have found that row orientation can suppress weed growth and increase crop yield [83]. Our results suggest that orientation can impact earlier development, but these differences can potentially reduce over time, especially if stands are not thinned in a timely manner once canopy growing space has diminished.

### Assessment of tree crown complexity indices and volume

We evaluated the ability of ITC 3D shape complexity and 3D alphashape volume to predict stem size and stem size changes. When OPCR and crown volume model coefficients were calculated per genotype, there were noticeable differences observed among values, implying differences in crown shape, size, and growth efficiency among genotypes, as was also found by Chmura et al. [71,84].

There were differences between the 2017 and 2021 dataset for both the OPCR complexity index and the alphashape volume, reflective of the growth of the crowns over time. We would expect crown shape to change as the tree grows. For example, Baldwin and Peterson [85] modeled the change from conical juvenile loblolly crowns to more ellipsoidal shapes as the tree matured.

It follows that a larger crown object may result in a more complex shape. This reasoning was supported by our results, where 3D alphashape crown volume and OPCR were correlated in linear models and exhibited significant interactions in mixed effects. When compared, OPCR and crown volume did not exhibit multicollinearity when predicting stem size and stem volume change, indicating that these variables were describing different features of the crown, size, and shape. Linear models (Table 11) containing only crown volume metrics generally had higher *R*^2^ and lower AIC values when estimating either stem volume or DBH change. Models that included both OCPR and crown volume metrics again generally outperformed models containing OCPR metrics alone. This implies that DLS-derived crown volume is more strongly related to stem size within this study. We divided a tree’s crown volume by its shape complexity index value to create a new metric (complexity per unit of volume), which was significantly related to stem volume and DBH change; however, it was only weakly correlated. This provides only a weak implication that complexity in crown shape adds much to the predictive power of this when relating crown metrics to stem growth.

### Tree crown volume in different azimuths

The 6 different genotypes exhibited statistically significant differences in crown volume for each of the 8 azimuths tested, with 2 exceptions. Two pairs of genotypes (1 and 5, and 2 and 6) were similar (*P* > 0.05) in crown volume across all tested azimuths. When plotted (Figs. 6 and 7), all genotypes appeared to have the largest proportion of crown volume change facing south–southeast. Over time, genotypes 2 and 6 comparatively had relatively little crown volume change/increase facing north. Genotypes 1, 3, and 5 generally had more uniform crown volume change, with a small tendency toward the south. Genotype 4, however, did not appear to alter its relative crown volume over time and was generally uniform among azimuths.

We also evaluated crown volume and crown volume change in the northern and southern directions and their potential effects on stem growth. Increases in the southern-crown segment volume generally represented more positive stem volume change per unit of crown volume change than the northern-crown segment. Both north- and south-crown segment volumes were significantly related to stem growth. Genotype did not significantly impact crown change in either direction. While there appears to be variation in crown size change by direction for each genotype (Fig. 7), these results would imply that there may be too much variability within each genotype to discern an affect. Note that wedge size was increased from 45° to 90° for this part of the study that may also increase the variability of volumes observed. Crown volume change and segment direction were significant when modeled against stem volume change. Within the context of the current study, these results imply that crown growth direction will influence stem growth, and suggest that growth in the southern-crown volume has a higher stem volume growth efficiency. Previous studies have implied that crown metrics (e.g., crown volume, crown length, and leaf area index) correlate with stem size (e.g., [38,86,87]). Thus, larger tree crowns will result in greater stem growth.

Rouvinen and Kuuluvainen [34] and Pretzsch [9] observed similar growth behavior in terms of crown asymmetry and plasticity with regard to the prevalent directionality of solar radiation in order to increase light interception. The presence of competing vegetation and the availability of gaps (caused by mortality or thinning operations) has also been linked to the causes of crown asymmetry (e.g., [9,34,82,88]). The prevailing row direction in the majority of our study site (75% were south-east) may explain the differences in volume in directions that are not purely to the south (i.e., the direction of maximum solar radiation exposure). The effects of the terrain’s slope on crown asymmetry has also been demonstrated [19] but assumed to be of little effect in our study. Given ALS ability to provide digital terrain models, future work could evaluate if this information would be of benefit.

Our results suggest that the 6 loblolly pine genotypes present at our study site exhibited differences in crown plasticity. Chmura et al. [71,84] noted that genotypes could exhibit differences in crown shape. High pulse density DLS allows us the potential to evaluate changes at scales within the ITC, and over large spatial extents, such as complete stands.

### Considerations and future work

The methods presented within this study have demonstrated that crown shape size and indices of complexity, as derived from alphashape fitting, are statistically significant variables when considering tree stem size change. The variability of ITC volume can also be assessed. There was variability in the loblolly pine genotypes included within our study resulting in observable differences in crown asymmetry and plasticity. Pretzsch [89] posited that the denser and more plastic canopy space filling in stands may increase light interception, stand density, productivity, and growth resilience to disturbances. DLS therefore provides a means of monitoring and predicting stand development over large scales.

Tree crown size and change is a key variable in some published modeling methods, which include PTAEDA4.0 [14] and TRULOB [3]. Lamb et al. [90] demonstrated that ALS-derived metrics can be used as inputs to such growth models. Sumnall et al. [78] demonstrated that competitive neighborhoods, of crown size metrics, could be calculated from DLS datasets for the estimation of stem size. These methods assume a simplified crown shape and do not typically consider the availability of growing space or gaps. The incorporation of improved estimates of crown shape and size into growth models, at the ITC scale, has the potential to improve estimates in contexts where crown size can vary (e.g., trees next to gaps or thinned rows, and in areas where slope is variable). Another potential application area is in power-line management where vertical and lateral growth of vegetation into specific 3D areas could be modeled to inform the timing and efficacy of management.

We compared 3D alphashape metrics derived from 2 separate DLS acquisitions. There were differences in tree size and pulse density between these acquisitions. DLS point density varies by depth within a canopy [41]. It is reasonable to assume that fewer returns were available from lower canopy elements, with fewer still if the overstory was composed of dense foliage due to occlusion. Variable pulse density and the constraints on the alphashape method in determining what the crown exterior is may have led to estimation error. For example, too large an α value would result in a shape approximating a convex hull larger than the tree crown and too small a value would potentially result in one or more shapes formed around only part of the point cloud [73]. Fitting 3D alphashape meshes, therefore, requires a sufficient number of returns to represent the exterior of the object, and a sufficient α value to form a single surface appropriate to the complexity and size of the object. Within the current study, we used an α value of 1 m for all classified ITCs and was assumed to create 3D meshes sufficient to characterize the shapes encountered. What alphashape settings to optimally characterize an ITC remain uncertain particularly as pulse density will vary by acquisition and height. Using laser scanning to characterize a tree’s crown, although imperfect, is still likely an improvement over the simplified approach often used in typical field-based methods. Likewise, the accuracy of the results is dependent on the ITC delineation method and its ability to classify returns correctly. In more structurally diverse forests, such as where tree crowns interlock, the ITC classification methodology would be of major concern.

The calculation of 3D alphashape metrics is dependent on the accurate classification of DLS returns from the ITC. Competing vegetation or interlocking branches in the canopy may cause issues in adapting this current approach directly to other contexts, as incorrectly classified returns would cause additional uncertainty in crown shape. Within the current study, we only assessed relatively wide spaced trees (618 trees per hectare) and for locations where there was no understory, thus reducing the likelihood of these conditions. Any future work should consider including different stem spacing and management conditions.

Determination of crown growth in different directions is dependent on being able to accurately locate the stem. Several studies have shown that crown centers differ from stem locations (e.g., [31,91,92]). The ability to resolve returns from lower canopy elements [41], and classify the stem, presents a number of challenges when considering the application of the current approach to different ALS acquisitions and lower pulse densities.

## Conclusion

In conclusion, DLS datasets have strong capabilities with regard to the characterization of 3D ITC shape, which can be applied to all ITCs over large areas. We presented a novel method to classify DLS returns into ITC objects, identify the location of the tree stem, and calculate 3D alphashape metrics from 2 temporally disparate DLS acquisitions and compare the changes in managed loblolly pine stands among 6 genotypes exhibiting different crown architectures. Our results demonstrate that DLS can be used to assess crown growth, shape asymmetry, and plasticity over time. Statistically significant differences were observed among the different genotypes, demonstrating developmental differences in crown asymmetry and exhibiting growth directionality related to light interception and the availability of growing space caused by row orientation. Specifically, row orientation appears to influence growth rate. Increases in crown volume growth was statistically related to stem volume increase. More specifically, increases in the volume of the southern portions of the crown indicated larger increases in stem volume, in contrast to the volume of the northern crown volume. Future research is warranted in further developing these approaches for other environmental contexts and DLS/ALS datasets, as these metrics could potentially improve our models for growth and yield predictions exploiting the greater potential of crown characterization that methods such as DLS provide, certainly in comparison to what is capable using typical field measurements.

## Data Availability

Data available on request from the authors. The data that support the findings of this study are available from the corresponding author, M.J.S., upon reasonable request.
